# Beneficial Effects of Infiltration of Platelet-Rich Plasma in the Endometrium

**DOI:** 10.3390/biology14040319

**Published:** 2025-03-21

**Authors:** Paula Alonso-Frías, Emilio Francés-Herrero, Clara Bueno-Fernandez, María Gómez-Álvarez, Marcos Agustina-Hernández, Irene Cervelló, Mauro Cozzolino

**Affiliations:** 1 IVIRMA Global Research Alliance, IVI Foundation, Instituto de Investigación Sanitaria La Fe (IIS La Fe), 46026 Valencia, Spain; paula.alonso@ivirma.com (P.A.-F.); emilio.frances@ivirma.com (E.F.-H.); maria.gomez@ivirma.com (M.G.-Á.); marcos.agustina@ivirma.com (M.A.-H.); irene.cervello@ivirma.com (I.C.); mauro.cozzolino@ivirma.com (M.C.); 2 Department of Pediatrics, Obstetrics and Gynecology, Faculty of Medicine, University of Valencia, 46010 Valencia, Spain; 3 IVIRMA Global Research Alliance, IVI Roma, 00197 Rome, Italy

**Keywords:** endometrium, platelet-rich plasma, fertility, molecular mechanisms, endometrial pathologies

## Abstract

Platelet-rich plasma (PRP) is a concentrated product of autologous plasma platelets. It promotes the repair of tissues with low healing potential by providing supraphysiological amounts of essential growth factors and has recently become more popular in endometrial repair, achieving exciting clinical results. PRP treatment has proven to improve fertility outcomes in patients with a poor endometrial environment. However, the mechanism is not yet clear. Previous preclinical models also showed that PRP treatment decreased the expression of inflammatory markers and fibrosis, increased the endometrial proliferation rate and gene expression, and enhanced the pregnancy rate. The modulation of the endometrial immune environment and endometrial microbial community by PRP treatment appeared to be the key mechanism by which it improved endometrial receptivity. This review summarized the potential of adult PRP based on its composition and applications and the biological mechanisms and biological modifications in the endometrium after PRP instillation in preclinical models.

## Simple Summary

This review focuses on the potential of platelet-rich plasma in improving poor endometrial proliferation and function. Platelet-rich plasma is a blood-derived product rich in growth factors that promote tissue healing. While its use in endometrial repair has shown promising preclinical results, the underlying mechanisms remain unclear. In this paper, we collect and detail the composition, method of obtaining, and the main mechanisms of action of platelet-rich plasma. We explain these results both in diverse tissues and specifically in the endometrium, which can help us understand the therapeutic effects this product has shown in human endometria.

## 1. Introduction

### 1.1. Platelet-Rich Plasma (PRP)

#### 1.1.1. PRP Definition and Classification

Platelet-rich plasma (PRP), also known as plasma rich in growth factors (PRGF), is defined as a biological product with a platelet concentration that exceeds baseline levels typically found in the blood of healthy individuals. In normal human blood, platelet counts range from 150,000 to 450,000 platelets per microliter (µL). During the preparation process, centrifugation of blood concentrates platelets within a reduced plasma volume, resulting in a final concentration of approximately 1 × 10^6^ platelets per µL. This elevated concentration qualifies the enriched preparation as PRP [1,2].

The source of PRP for the different applications can vary, encompassing autologous; when it comes from the same individual, allogenic; if the PRP is obtained from another subject of the same species or heterologous; when it comes from another species.

There have been other classification systems proposed besides source-based classification. The earliest one, developed by Ehrenfest et al., categorized PRP according to leukocyte and fibrin content into four distinct types: pure platelet-rich plasma (P-PRP), leukocyte- and platelet-rich plasma (L-PRP), pure platelet-rich fibrin (P-PRF), and leukocyte- and platelet-rich fibrin (L-PRF) [3]. Subsequent classifications introduced criteria such as platelet concentration [4] and activation status [5].

The most recent classification, recommended by the Society for Thrombosis and Hemostasis and endorsed by the Scientific Committee on Standardization, integrated prior classifications and categorized PRP based on leukocyte percentage, red blood cell content, preparation method, activation method, and platelet concentration [6].

The diversity of classification systems arises from the lack of standardization in PRP preparation methods, leading to variability in cell types, biomolecules, growth factors (GFs), cytokine content, and release times [7].

While there is no consensus on the precise definition of well-prepared PRP, the literature outlines certain criteria, including a platelet concentration 4 to 6 times greater than the baseline level in peripheral blood, the absence of leukocytes or counts below 1000/mL, and erythrocytes at concentrations equal to or less than 100/mL. However, exceeding a platelet concentration of six times the baseline is not recommended, as evidence suggests that this may diminish the therapeutic efficacy [2].

#### 1.1.2. PRP Preparation Protocols

Regardless of the variations in PRP preparation protocols, they all begin with a peripheral blood draw of approximately 20–60 mL into a system that contains an anticoagulant, such as citrate dextrose A (ACD-A), sodium citrate, or ethylenediaminetetraacetic acid (EDTA) [8]. From this point, two preparation methods can be employed: the open and closed techniques. In the open technique, blood is exposed to air at some stage of the process, which may increase the risk of contamination. In contrast, the closed technique utilizes commercial devices to prevent air exposure, thereby minimizing contamination risks [1].

**Figure 1:**
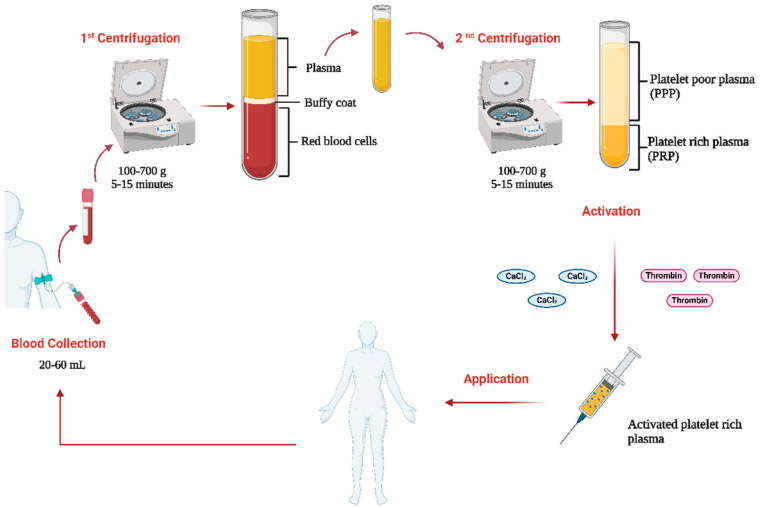
Preparation protocol of human PRP. The figure shows the process of preparing Platelet-Rich Plasma (PRP). First, 20–60 mL of peripheral blood is collected from the patient/donor and subjected to a first centrifugation (100–700 g for 5–15 min) to separate the blood into three layers: plasma at the top, a platelet-rich intermediate layer (buffy coat), and red blood cells at the bottom. Then, the plasma and the intermediate layer are subjected to a second centrifugation, which produces two fractions: platelet-poor plasma (PPP) and PRP. The PRP is activated by adding calcium chloride (CaCl₂) or thrombin, facilitating the release of GFs. Finally, the activated PRP is applied to the patient via injection to promote tissue regeneration and accelerate healing. CaCl_2_: Calcium chloride. Created with https://www.biorender.com/ (accessed on 3 November 2024).

In both methods, the speeds range between 100 and 700 g, with centrifugation times typically between 5 and 15 min (Figure 1). One or two centrifugations can be used, with double centrifugation being recommended as it more effectively concentrates the platelets [9]. In the first centrifugation, blood is divided into three layers: the bottom layer is composed of red blood cells, the middle white buffy coat is composed of leukocytes, and the top layer is composed of plasma and platelets. Usually, the top layer is centrifuged a second time at a higher speed, separating the plasma in a supernatant that corresponds with platelet-poor plasma (PPP) and a pellet where the platelets are concentrated corresponding to the PRP [10]. PRP is usually activated prior to its application using calcium chloride (CaCl_2_) or thrombin. However, in some cases, it is preferred to directly inject it into the tissue allowing contact with collagen to activate the release of platelet granules [7].

### 1.2. Clinical Applications

Besides the different protocols employed to prepare PRP, this blood derivative has been used in several medical fields such as dermatology, orthopaedics, odontology, ophthalmology, and cardiac injuries among others [11].

PRP began to be applied around 1920 as a treatment for patients with thrombocytopenia; however, the term “platelet-rich plasma” was not coined until 1954 by Kingsley [12]. In the following decades, its use gained widespread popularity, and in 1972, it was first employed to regulate hemostasis during surgical procedures [13]. By 1980, PRP had been implemented in various types of surgeries, and the characterization of the GFs it contains was starting [14,15]. For instance, maxillofacial surgery was one of the first to incorporate the use of PRP at the beginning of this decade improving bone regeneration [16].

In recent decades, several preclinical models have been developed to investigate the potential of PRP as an effective therapy in cardiology. These studies have shown promising results, including neovascularization in sheep following PRP application [17] and high tissue regeneration in the infarcted areas of rabbits [18].

In dermatology, PRP has been used since the late 1990s, particularly for facial rejuvenation, due to its ability to increase elastic fibres and stimulate collagen production [19]. It is commonly employed in the treatment of scars, burn wounds, and skin repigmentation in conditions such as vitiligo [20]. In orthopaedics, PRP gained popularity in the early 2000s, especially for tendon injuries and meniscus tears. However, its effectiveness in treating osteoarthritis and tendinopathies remains inconsistent [21]. In dentistry, PRP began to be used in the 1990s and has shown efficacy in gingival depigmentation, dental implants, antimicrobial infections, and regenerative endodontic treatments [22]. In ophthalmology, PRP is used to treat macular holes and improve patient health in conditions such as dry eye and retinitis pigmentosa [23]. A variant of PRP known as Eye-PRP, which is enriched with specific growth factors, is commonly used in ophthalmic practice [24].

In addition to its established medical applications, PRP is now being utilized in the management of spinal disorders, pain management, and reproductive medicine. In reproductive medicine, PRP is applied for ovarian rejuvenation and in cases of thin endometrial lining [25].

### 1.3. Reproductive Medicine Applications

In this discipline, PRP is primarily being studied to address ovarian conditions like poor ovarian reserve or premature ovarian insufficiency, as well as endometrial pathologies such as thin or atrophic endometrium (TE), recurrent implantation failure (RIF) and Asherman syndrome (AS) [25].

The endometrium is the inner lining of the uterus, which thickens during each menstrual cycle in preparation for potential embryo implantation. It plays a crucial role in fertility; as a receptive endometrium is essential for successful pregnancy. Even when the embryo is healthy, a non-receptive endometrium can prevent the early stages of pregnancy from progressing or hinder the embryo’s attachment to the uterine lining [26]. A non-receptive endometrium is associated with implantation failure and early pregnancy loss, which leads to reduced pregnancy rates in in vitro fertilization (IVF). This makes it a critical factor to consider in reproductive health and fertility treatments [27]. A non-receptive endometrium may be caused by TE, where the lining is insufficiently thick for embryo implantation (<7 mm), or by endometrial scarring or AS, where the endometrium is damaged or has adhesions that reduce the implantation sites for the embryo [28].

Conventional approaches for these pathologies include hormonal treatments such as oestrogen therapy, vasodilators like aspirin, and surgery to assess uterine adhesions [29]. While these therapies can improve conditions, their success varies between patients, as individual responses to treatment can differ significantly, often resulting in low effectiveness [30].

This is why novel therapies based on regenerative medicine, such as PRP, are gaining popularity, as they lead to improved endometrial thickness, blood flow, and implantation rates [31]. Studies report that women with TE treated with autologous PRP often experienced an increase in endometrial thickness [32] and improved pregnancy outcomes in IVF cycles [33]. These results are aligned with clinical findings on PRP hysteroscopy injection in women with TE, demonstrating a rise in endometrial thickness and enhanced local microcirculation [34].

In cases of AS, PRP has recently emerged as an alternative treatment. Available evidence shows that PRP not only enhances endometrial growth [35] but also increases pregnancy success in these patients [36,37].

Additionally, PRP may be a therapeutic option for women who experience RIF. The available evidence suggests that it could improve reproductive outcomes in these patients. However, the limited number of studies, differences between protocol preparations, activation methods, quantities, injection types, and small sample sizes contribute to somewhat inconsistent results [38].

Findings suggest a potential role of PRP in enhancing endometrial receptivity and as a therapeutic option for women who suffer from TE, AS, or RIF [38]. However, despite these positive results, the mechanism by which PRP exerts its effects on the endometrium is not yet fully understood. While PRP is rich in GFs, which are believed to stimulate tissue repair and regeneration, the exact biological pathways and molecular mechanisms involved remain unclear and require further research.

In addition, PRP treatment protocols are not yet fully standardized due to a lack of studies evaluating the most effective method of application or the optimal number of applications required for treating endometrial pathologies. These protocols vary between injection under hysteroscopy and perfusion or instillation via a uterine catheter guided by ultrasound. Moreover, the number of instillations also differs across studies [30].

For these reasons, further research is required to establish standardized guidelines and optimize the therapeutic use of PRP in endometrial treatments, aiming to clarify the most effective protocol for preparation and application, as well as the mechanism of action of this therapy.

This review describes the identified PRP mechanisms of action in the endometrium and seeks to elucidate its possible relationship with endometrial and fertility outcomes in various clinical trials.

## 2. Materials and Methods

P.A-F., E.F-H., and C.B-F. conducted a search of PRP and endometrium available in PubMed and Google Scholar (supervised by I.C. and M.C.). The search was limited to full-text articles published in English until November 2024. The following keywords were applied: PRP, endometrium, inflammation, angiogenesis, and growth factors. When the full texts were not available, a request was sent to the corresponding author(s). Additional studies were identified by manually searching the references of selected articles and complementary reviews.

## 3. Research Findings

### 3.1. PRP Composition

The composition of adult PRP may vary depending on the preparation method used and the individual’s physiological characteristics, leading to different concentrations of leucocytes and erythrocytes in the final product. Regardless of this variation, platelets are the main and key component of PRP [1].

**Figure 2:**
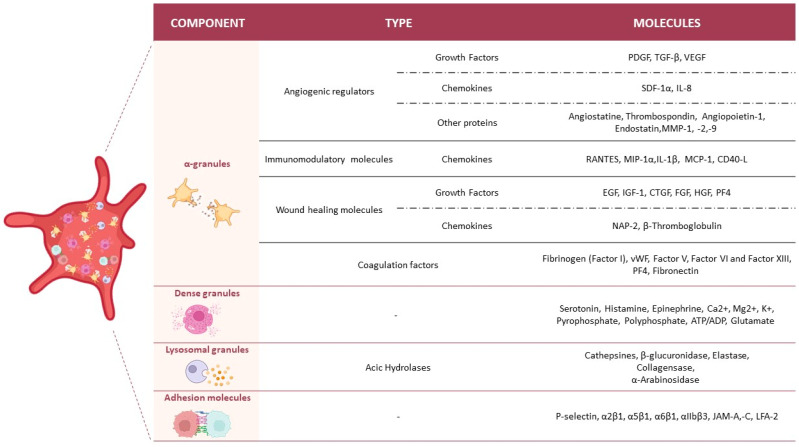
Platelet granules and their content. The table summarizes the key molecules found in platelets, categorized by their specific granules and adhesion molecules. Alpha (α)-granules contain angiogenic regulators such as growth factors (e.g., PDGF, TGF-β, VEGF) [41], chemokines (e.g., SDF-1α, IL-8) [13] and other proteins with this angiogenic effect (e.g., angiostatin, thrombospondin, angiopoetin, endostatin and MMP -1,-2,-9) [42]. Immunomodulatory chemokines (e.g., RANTES, MIP-1α, IL-1β, MCP-1, CD40-L) [13,39], wound healing molecules such as growth factors (e.g., EGF, IGF-1, CTGF, FGF, HGF, PF4) [3,39], and chemokines (e.g., NAP-2, β-Thromboglobulin) are also present in these granules [41]. They also contain coagulation factors (e.g., fibrinogen, vWF, Factor V, Factor VI, Factor XIII, PF4, fibronectin) [39]. Dense granules store small molecules such as serotonin, histamine, epinephrine, Ca^2^⁺, Mg^2^⁺, K⁺, pyrophosphate, polyphosphate, ATP/ADP, and glutamate [13,40,43]. Lysosomal granules contain acid hydrolases such as cathepsins, β-glucuronidase, elastase, collagenase, and α-arabinosidase [44]. Adhesion molecules such as P-selectin, α2β1, α5β1, α6β1, αIIbβ3, JAM-A, JAM-C, and LFA-2 play essential roles in platelet interaction with other cells and the extracellular matrix [45,46]. ATP/ADP: Adenosine Triphosphate/Adenosine Diphosphate; Ca^2+^: Calcium Ion; CD40-L: CD40 Ligand; CTGF: Connective Tissue Growth Factor; EGF: epidermal growth factor; FGF: fibroblast growth factor; HGF: hepatocyte growth factor; IGF-1: insulin-like growth factor 1; IL-1β: Interleukin 1 Beta; IL-8: Interleukin 8; JAM-A, JAM-C: Junctional Adhesion Molecule A, C; K^+^: Potassium Ion; LFA-2: Lymphocyte Function-associated Antigen 2; MCP-1: monocyte chemoattractant protein 1; Mg^2+^: Magnesium Ion; MIP-1α: Macrophage Inflammatory Protein 1 Alpha; MMP-1, -2, -9: Matrix metalloproteinase 1, 2, and 9; NAP-2: Neutrophil Activating Peptide 2; PDGF: platelet-derived growth factor; PF4: platelet factor 4; RANTES: regulated upon activation, normal T cell expressed and secreted; SDF-1α: stromal cell-derived factor 1 alpha; TGF-β: transforming growth factor beta; VEGF: vascular endothelial growth factor; vWF: von Willebrand factor. Created with https://www.biorender.com/ (accessed on 3 November 2024).

Platelets are anucleate cells that derive from megakaryocytes in the bone marrow. They play a crucial role in hemostasis, preventing blood loss during vascular injury by creating a clot. The processes involved are platelet adhesion to the subendothelial matrix, and platelet activation to promote degranulation and the secretion of signalling molecules and GFs that enhance the stabilization of the clot and wound healing. The therapeutic effects of PRP in tissue are largely attributed to these GFs, which stimulate angiogenesis and tissue repair [39]. To carry out these functions, platelets contain three main kinds of granules: α-granules, dense granules, and lysosomal granules [40] (Figure 2).

α-granules represent 10% of the platelet volume and contain large polypeptides involved in several functions, including coagulation, inflammation, angiogenesis, wound healing, and immunological defence. They mainly concentrate on coagulation factors, a group of proteins that work together in a complex cascade to synthesize the fibrin clot. Key coagulation factors present in α-granules are fibrinogen (Factor I), von Willebrand factor (vWF), Factor V, Factor VI, and Factor XIII [39].

Within α-granules, we also find immunomodulatory molecules that regulate the immune response by enhancing (immunostimulatory) or suppressing (immunosuppressive) immune system activity. Additionally, these granules contain angiogenic regulators that promote or inhibit new blood vessel formation (angiogenesis). Both groups include GFs, cytokines, chemokines, and other signalling molecules [41].

The GFs stored in the α-granules are released upon platelet activation. These factors are proteins that stimulate cellular proliferation, differentiation, and survival by binding to specific receptors on the surface of target cells, triggering intracellular signalling pathways that lead to specific cellular responses. Some of the most important GFs are platelet-derived growth factor (PDGF), transforming growth factor-beta (TGF-β), and vascular endothelial growth factor (VEGF) [47].

Another essential component of the α-granules is chemokines, which direct immune cells migration to sites of inflammation or injury by binding to specific receptors on their surfaces. These chemokines include C-C motif chemokine ligands (CCL), which primarily recruit monocytes, macrophages, and T cells. Notable examples include CCL5 (regulated upon activation, normal T cell expressed and secreted, or RANTES), CCL2 (monocyte chemoattractant protein-1, or MCP-1), and CCL3 (macrophage inflammatory protein-1 alpha, or MIP-1α). In addition to CCLs, C-X-C motif chemokine ligands (CXCL) are also present, which attract neutrophils and stem cells. Key examples include CXCL4 (platelet factor 4, or PF4), CXCL12 (stromal cell-derived factor 1 alpha, or SDF-1α), and CXCL8 (interleukin-8, or IL-8) [13].

The second type of granule present in platelets is dense granules. Unlike α-granules, dense granules are less abundant and concentrate few proteins and fewer types of small molecules. Their main action is to accelerate platelet activation and contribute to immune response [13]. Key constituents of dense granules crucial in the platelet activation process are serotonin, histamine, epinephrine Ca^2+^, Mg^2+^, K^+^, pyrophosphate, polyphosphate, and a pool of non-metabolic adenine nucleotides such as ADP and ATP. These components are also recognized by receptors on dendritic cells, developing immune cell-modifying effects [43].

The third type of platelet granules is lysosomal granules, named for their similarity to lysosomes. These granules are characterized by an acidic interior containing acid hydrolases, various enzymes, and lysosomal membrane markers [48]. Acid hydrolases include enzymes such as proteases, glycosidases, and phosphatases, which degrade proteins, lipids, carbohydrates, and nucleic acids. Other notable enzymes in these granules include proteases, such as cathepsins involved in tissue repair, and β-glucuronidase, which degrades glycosaminoglycans during the remodelling of the extracellular matrix (ECM) [44].

Finally, platelets possess adhesion proteins that initiate the response during hemostasis. These proteins mediate the interactions between platelets and the ECM, facilitating platelet aggregation and adhesion during clot synthesis [49]. P-selectin is a protein that promotes platelet-endothelial and platelet-leukocyte interactions. Other adhesion molecules mainly belong to two integrin families: β1 integrins (α2β1, α5β1 and α6β1) and β3 integrins (αIIbβ3) [45,50].

### 3.2. Mechanisms of PRP

**Figure 3:**
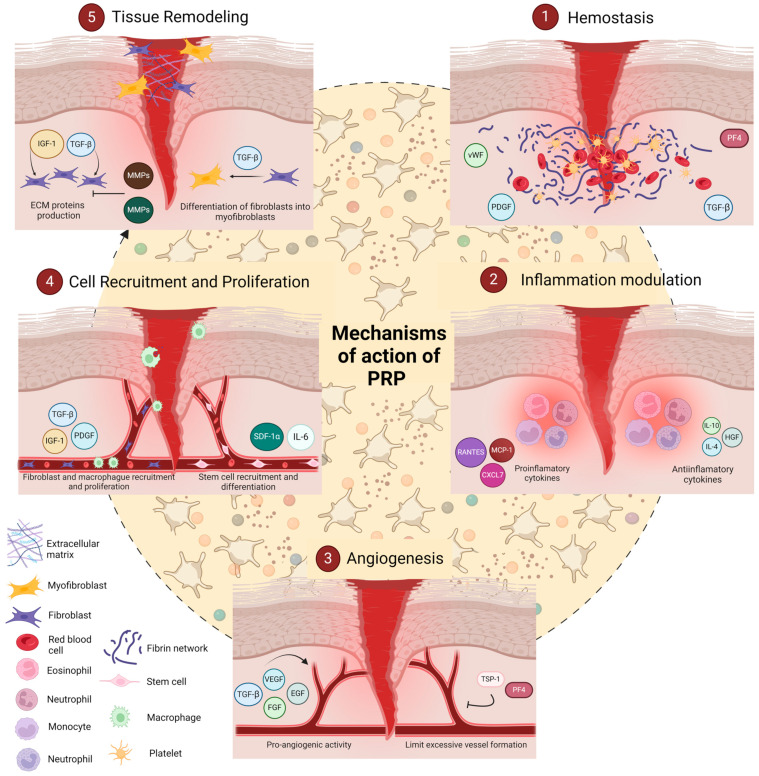
Mechanisms of action of PRP in tissue repair and regeneration. The figure illustrates the multifaceted biological processes mediated by PRP through five interconnected mechanisms: (1) hemostasis: PRP promotes the stabilization of the wound site by releasing key factors, such as PF4, vWF, PDGF, and TGF-β, facilitating clot formation and initiating the healing cascade. (2) Inflammation modulation: PRP regulates the inflammatory response by balancing pro-inflammatory cytokines (e.g., RANTES, MCP-1, and CXCL7) and anti-inflammatory mediators (e.g., IL-10, IL-4, and HGF), contributing to an environment conducive to healing. (3) Angiogenesis: PRP enhances new blood vessel formation via pro-angiogenic factors such as VEGF, FGF, EGF, and TGF-β, while also preventing excessive vascularization through anti-angiogenic regulators like TSP-1 and PF4. (4) Cell recruitment and proliferation: PRP supports the recruitment and proliferation of fibroblasts, macrophages, and stem cells by releasing TGF-β, IGF-1, PDGF, SDF-1α, and IL-6, essential for tissue repair and regeneration. (5) Tissue remodelling: PRP drives ECM remodelling and scar maturation by inducing MMPs, promoting fibroblast differentiation into myofibroblasts, and enhancing the production of ECM proteins through growth factors like TGF-β and IGF-1. CXCL: chemokine C-X-C motif ligand 7; EGF: epidermal growth factor; FGF: fibroblast growth factor; HGF: hepatocyte growth factor; IGF-A: insulin-like growth factor A; IGF-1: insulin-like growth factor 1; IL-4: interleukin 4; IL-6: interleukin 6; IL-10: interleukin 10; MCP-1: monocyte chemoattractant protein 1; MMPs: matrix metalloproteinases; PDGF: platelet-derived growth factor; PF4: platelet factor 4; RANTES: regulated on activation normal T cell expressed and secreted; SDF-1α: stromal cell-derived factor 1 alpha; TGF-β: transforming growth factor beta; TSP-1: thrombospondin-1; VEGF: vascular endothelial growth factor; vWF: von Willebrand factor. Created with https://www.biorender.com/ (accessed on 3 November 2024).

The mechanisms by which PRP enhances tissue healing are multifaceted and include (1) hemostasis, which establishes initial wound stability through clot formation; (2) inflammation and modulation, where PRP regulates the immune response to balance pro- and anti-inflammatory signals; (3) angiogenesis, where PRP promotes new blood vessel formation to supply nutrients and oxygen to the healing tissue; (4) cell recruitment and proliferation, which attracts and activates cells necessary for tissue repair; and (5) tissue remodelling, the final phase in which PRP aids in reorganizing and strengthening the newly formed tissue (Figure 3).

Together, these five mechanisms reflect the coordinated action of PRP’s molecular components in supporting each stage of the healing cascade, from initial stabilization to complete tissue restoration [46]. This review will delve into each of these processes, detailing how PRP orchestrates these mechanisms to achieve effective and efficient healing.

#### 3.2.1. Hemostasis

Hemostasis is the initial step in the tissue repair process, where PRP plays a crucial role in stopping bleeding and creating a structural matrix for cellular migration and attachment. Upon activation, platelets in PRP release essential haemostatic factors and initiate clot formation, stabilizing the wound and forming a provisional ECM scaffold that facilitates subsequent healing stages [51,52].

PRP’s haemostatic action begins with platelet aggregation, mediated by glycoprotein receptors (GPIIb/IIIa), which bind fibrinogen and vWF, enhancing platelet adhesion to the injury site. Upon binding, platelets release thrombin and calcium ions, converting fibrinogen into fibrin, resulting in a mesh that stabilizes the wound [53]. This fibrin network acts as an ECM scaffold, providing structural integrity and guiding the migration of fibroblasts and endothelial cells involved in tissue repair [54].

In parallel, activated platelets release a suite of growth factors critical to initiating the repair process. Among these, PDGF and TGF-β play prominent roles. PDGF recruits fibroblasts and smooth muscle cells to the wound, restoring structural integrity, while TGF-β promotes collagen synthesis and ECM stabilization [55]. Additionally, PF4 and serotonin contribute to vasoconstriction, reducing blood flow to the injured area [56].

#### 3.2.2. Inflammation Modulation

The inflammatory phase is a critical part of the healing process, preparing the injury site for tissue repair by managing immune responses and clearing damaged cells. PRP aids in inflammation modulation by balancing pro- and anti-inflammatory signals, preventing excessive inflammation while still promoting healing [57].

PRP releases chemokines such as CXCL7, MCP-1, and RANTES, which attract neutrophils and monocytes to the wound [58]. These cells initiate a pro-inflammatory environment that clears debris and prevents infection. CXCL7, in particular, promotes neutrophil migration, while MCP-1 and RANTES support monocyte and T-cell infiltration, critical for early immune response [59,60].

To prevent prolonged inflammation, PRP contains cytokines such as IL-4, IL-10, and hepatocyte growth factor (HGF), which shift the immune response toward an anti-inflammatory profile. IL-10 downregulates pro-inflammatory cytokines (e.g., IL-1 and TNF-α), while IL-4 aids in transitioning macrophages from the inflammatory M1 phenotype to the healing M2 phenotype, which releases factors that facilitate tissue repair [61,62]. In addition, HGF modulates the nuclear factor kappa B (NF-κB) signalling pathway, reducing inflammation by inhibiting NF-κB activation. PRP lowers the expression of pro-inflammatory genes, which helps control pain and reduces catabolic activity in surrounding tissues. This effect is particularly relevant in chronic inflammatory conditions, where NF-κB overactivation contributes to tissue degradation [63].

#### 3.2.3. Angiogenesis

Angiogenesis, the formation of new blood vessels, is essential for supplying injured tissue with oxygen and nutrients, ensuring effective healing. PRP promotes angiogenesis by releasing a combination of pro-angiogenic factors, including VEGF, fibroblast growth factor (FGF), epidermal growth factor (EGF), and TGF-β [42,64].

VEGF and EGF are the primary drivers of angiogenesis in PRP. VEGF promotes endothelial cell proliferation and migration, enabling new capillaries to form at the injury site. EGF complements this action by stimulating endothelial cell mitogenesis, establishing a vascular network that supports the healing tissue [64]. The hypoxic environment of an injured site naturally triggers an angiogenic response and PRP amplifies this response by delivering concentrated angiogenic signals, which, in hypoxic conditions, promote additional VEGF release. This mechanism is particularly beneficial in treating ischemic injuries, where increased blood flow is critical for recovery [65].

Importantly, PRP maintains a delicate balance between promoting and controlling blood vessel growth. Pro-angiogenic molecules such as VEGF and FGF are counterbalanced by factors like thrombospondin-1 (TSP-1) and PF4, preventing excessive vessel formation. This balance is essential to avoid pathological angiogenesis, which could lead to scarring or abnormal tissue growth [66].

#### 3.2.4. Cell Recruitment and Proliferation

PRP not only modulates inflammation and angiogenesis but also actively stimulates cell proliferation, essential for tissue formation and repair. Key growth factors within PRP, such as PDGF and insulin-like growth factor 1 (IGF-1), act as chemotactic signals that attract fibroblasts to the injury site, promoting new tissue growth [67].

PDGF is a potent chemoattractant for macrophages, neutrophils, fibroblasts, and smooth muscle cells, all crucial for structural repair. Similarly, IGF-1 promotes fibroblast migration to the injury site, where they can proliferate and contribute to new tissue synthesis [67]. Once recruited, these cells require growth signals to proliferate and differentiate into tissue-specific cells. FGF and TGF-β are essential in this phase, promoting mitosis and cell differentiation, respectively. These factors stimulate fibroblasts and other progenitor cells to enhance collagen production, a key ECM structural component [58].

Interestingly, PRP’s SDF-1α content aids in the recruitment of stem cells to the wound. This factor, along with cytokines like IL-6, enhances repair potential by activating progenitor cells that can differentiate and regenerate damaged tissue, making it a promising mechanism in regenerative medicine applications [54].

#### 3.2.5. Tissue Remodelling

Tissue remodelling, the final phase of the healing cascade, involves ECM reorganization and stabilization, crucial for restoring tissue structure and function. PRP supports this phase by regulating anabolic and catabolic processes within the ECM, mediated by growth factors and matrix-modifying enzymes. Through the modulation of ECM production and degradation, PRP ensures that newly formed tissue is durable and functional [58,68].

Transforming growth factor-beta (TGF-β) and insulin-like growth factor-1 (IGF-1) play pivotal roles in promoting ECM synthesis during tissue remodelling. TGF-β stimulates fibroblasts to produce collagen, fibronectin, and other ECM proteins that strengthen the tissue, while IGF-1 enhances collagen deposition and stabilises ECM structure, crucial for long-term tissue integrity [52,69]. PRP also balances ECM remodelling by regulating matrix metalloproteinases (MMPs) and tissue inhibitors of metalloproteinases (TIMPs). MMPs degrade ECM components, allowing for tissue reorganization, while TIMPs counterbalance this activity to prevent excessive matrix breakdown. By modulating MMP and TIMP activity, PRP maintains an optimal balance between ECM synthesis and degradation, essential for functional tissue repair [70,71,72].

To aid tissue remodelling, PRP promotes fibroblast differentiation into myofibroblasts, specialized cells that produce collagen and exert contractile forces to close the wound. TGF-β and several interleukins present in PRP are crucial for myofibroblast differentiation and collagen maturation. This cell type plays a major role in wound contraction and collagen fibre reorganization, essential for achieving tensile strength in the newly formed tissue [55,62].

### 3.3. Mechanisms of Action in the Endometrium

#### 3.3.1. Preclinical In Vivo and In Vitro Models

Having established the broad therapeutic potential of PRP through its various biological actions, such as promoting tissue healing, modulating inflammation, and enhancing angiogenesis, it is crucial to delve into its specific mechanism in the endometrium. In vivo preclinical models have provided detailed insights into the specific biological processes responsible for endometrial function restoration.

**Figure 4:**
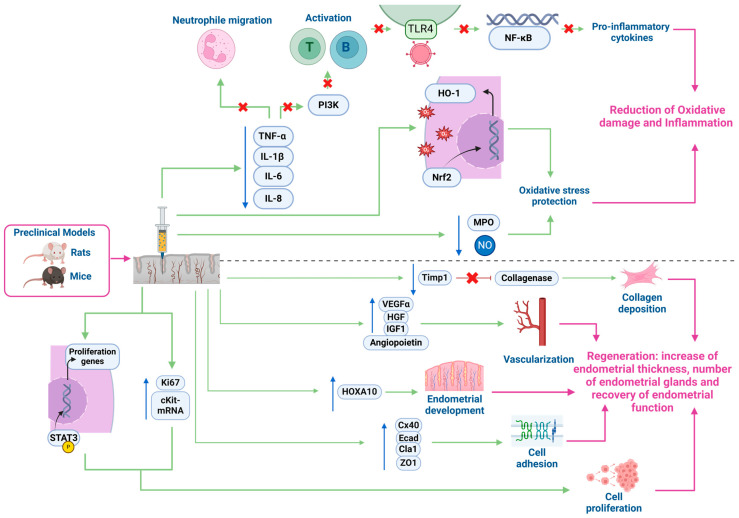
Endometrial action of PRP in in vivo models of damaged endometrium. The figure describes the mechanisms of action of PRP in inflammatory and regenerative processes. On the top, the reduction in oxidative damage and inflammation is illustrated. The activation of Nrf2 and HO-1 by PRP protects against oxidative stress. PRP also reduces the secretion of proinflammatory cytokines such as TNF-α, IL-1β, IL-6, and IL-8, and regulates the production of nitric oxide (NO) and other antioxidant molecules (MPO). Below, endometrial regeneration is highlighted. PRP stimulates cell proliferation through the phosphorylation of STAT3 and proliferation-related genes (such as Ki67 and cKit-mRNA). It promotes cell adhesion by regulating proteins like Cx40, E-cadherin (Ecad), Cla1, and ZO1, improving cell cohesion. Additionally, it enhances endometrial development and vascularization through factors such as VEGF, HGF, IGF1, and angiopoietin. It also supports collagen deposition by modulating collagenase and Timp1. Together, these effects increase endometrial thickness and the number of endometrial glands, and restore endometrial function. Finally, it is noted that these effects have been studied in preclinical models of rats and mice, reinforcing its potential in regenerative therapies. Cx40: Connexin 40; Cla: Claudin-1; cKit-mRNA: c-Kit tyrosine kinase receptor messenger RNA; Ecad: E-cadherin, HGF: hepatocyte growth factor; HO-1: heme oxygenase 1; HOXA10: Homeobox A10; IGF1: insulin-like growth factor 1; IL-1β: Interleukin 1 beta; IL-6: Interleukin 6; IL-8: Interleukin 8; Ki67: Ki-67 nuclear proliferation antigen gen; MPO: Mieloperoxidase; NF-κβ: Nuclear Factor Kappa B; NO: nitric oxide; Nrf2: nuclear factor erythroid 2-related factor 2; PI3K: Phosphatidylinositol 3-kinase; STAT3: Signal Transducer and Activator of Transcription 3; Timp1: Tissue Inhibitor of Metalloproteinases 1 gen; TLR4: toll-like receptor 4; TNF-α: tumour necrosis factor-alpha; VEGFα: vascular endothelial growth factor-alpha; ZO1: zona occludens-1. Created with https://www.biorender.com/ (accessed on 3 November 2024).

Recent in vivo studies have begun to reveal how PRP influences endometrial function and regeneration, revealing a promising avenue for enhancing fertility outcomes (Figure 4). The results from these preclinical models of endometrial damage align with in vitro studies conducted on different endometrial cell lines, including Ishikawa cells, endometrial stromal fibroblasts, and endometrial mesenchymal stem cells (MSCs). In these models, PRP exposure has been shown to produce a higher cell proliferation rate, a reduction in the expression of inflammation markers, and the upregulation of genes involved in tissue remodelling [73].

Particularly, preclinical animal models have proved that PRP exerts an anti-inflammatory effect on inflamed endometria. Studies on preclinical animal models of endometritis induced by lipopolysaccharide (LPS) and treated with PRP have indicated that PRP reduces the inflammatory response by lowering levels of myeloperoxidase (MPO), nitric oxide (NO), and proinflammatory chemokines such as tumour necrosis factor-α (TNF-α), interleukin-1β (IL-1β), interleukin-6 (IL-6), and interleukin-8 (IL-8) [74,75].

MPO catalyses the production of reactive oxygen species, while NO acts as a signalling molecule in inflammatory processes. Excessive MPO activity or high levels of NO can contribute to tissue damage and exacerbate inflammation due to oxidative stress. These chemokines also play a crucial role in promoting inflammation and are associated with chronic inflammation. TNF-α and IL-1β are mainly secreted by macrophages and promote the activation of other immune cells, leading to the production of additional inflammatory cytokines and the recruitment of immune cells to inflammation sites. IL-6 and IL-8, produced by various cell types such as endothelial cells, fibroblasts or immune cells, participate in T-cell and B-cell activation and in neutrophil infiltration to sites of inflammation. Notably, PRP has demonstrated a significant reduction in neutrophil infiltration and activation, which, if excessive, can lead to tissue damage due to the release of enzymes and reactive oxygen species [75].

To explain these results, various signalling pathways have been evaluated. PRP was able to reduce the inflammatory response to LPS by depressing the toll-like receptor 4/nuclear factor κB (TLR4/NF-κB) pathway and increasing the Nrf2/HO-1 pathway activity in endometrial cells and mouse endometrium. The TLR4/NF-κB pathway detects pathogens and triggers inflammation. When toll-like receptor 4 (TLR4) recognizes molecules from pathogens, it activates the NF-κB protein by releasing it from its inhibitor. NF-κB then enters the cell nucleus, promoting the recruitment of immune cells to the infection site by the production of pro-inflammatory cytokines and chemokines [75]. On the other side, the Nrf2/HO-1 pathway is a protective mechanism against oxidative stress and inflammation. When cells face stress, Nrf2 is activated and moves to the nucleus, where it promotes the expression of antioxidant genes like heme oxygenase 1 (HO-1). HO-1 then breaks down heme, the component of haemoglobin that can be toxic if it accumulates, into compounds that ameliorate oxidative damage and inflammation [76].

Beyond its anti-inflammatory effects, PRP has also been studied for its regenerative potential in the endometrium. Animal studies have proved that PRP ameliorated collagen deposition in preclinical models of ethanol-damaged endometrium in rats [77] and in mice affected by AS treated with human PRP [78,79].

Kshersagar et al. identified different markers related to healing processes that presented an elevated expression after PRP treatment in rats. These included alpha-smooth muscle actin (α-SMA), a marker of myofibroblast differentiation which is critical in wound healing and tissue repair; cell adhesion markers like connexin 40 (Cx-40), E-cadherin (E-Cad), claudin-1 (Cla-1), and zona occludens-1 (ZO-1), involved in cell communication and maintaining tissue integrity; and epithelial markers such as cytokeratin 18 and 19 (CK18, CK19) [78]. These results align with those presented by Jang et al., who also found a higher expression of regeneration markers in PRP groups like proliferation marker Ki-67; homeobox A10 (HOXA10), involved in endometrial development and c-Kit mRNA, crucial in cell growth, differentiation and survival. Other interesting markers identified were VEGF, a crucial factor in angiogenesis that had a higher expression than the control group, and IL-1β mRNA, a proinflammatory cytokine with a lower expression in the PRP group [78].

Expressions of various regeneration markers are consistent with findings provided by Kshersagar et al., where PRP contributed to an increase in glandular cell proliferation and a significant improvement in endometrial thickness in a rat model of ethanol-induced disturbed endometrium treated with PRP [78]. Additionally, in the control group, all rats were able to conceive, as well as all the PRP-treated rats. By contrast, any of the non-treated rats with disturbed endometrium gave birth to any live pups. These findings suggest that PRP treatment could not only regenerate damaged endometria but also lead to recovery of endometrial function with an improvement in endometrial receptivity [76].

Kim et al. also studied fibrotic markers in mice affected by AS treated with human PRP, reporting less expression of tissue inhibitor of metalloproteinase (Timp1), which inhibits collagenase and TGF-β1 that develops fibrosis by increasing ECM deposition [79]. Tissue remodelling of the mice endometria was also found in the h-PRP-treated group by significantly increasing the expression of genes involved in tissue remodelling, such as membrane type 2 matrix metalloproteinase gen (Mt2-MMP), which degrades ECM and activates other MMPs; lysil oxidase gen (Lox), which cross-links collagen and elastin to stabilize the ECM; and adrenomedullin gen (Adm), which induces vasodilation and regulates blood flow [78].

Furthermore, PRP demonstrated pro-angiogenic effects by significantly enhancing the expression levels of proangiogenic factors such as HGF, IGF-1, VEGF-α, and angiopoietin in treated mice, along with a lower expression of TNF-α compared with the non-treated group. This reduction in fibrosis and improvement of endometrial remodelling can be explained by a significant augmentation of STAT3 phosphorylation in mice treated with PRP. This is a key activation step that allows STAT3 to regulate genes that control cell growth, survival, inflammation, and immune responses by binding to DNA. Finally, PRP contributed to restoring fertility in AS mice, as all subjects in the treated group were able to deliver and had a shorter time to conceive with respect to the non-treated group where any of the mice were able to deliver [78].

Despite compelling evidence of positive PRP effects on inflammation and endometrial tissue regeneration, these studies have primarily been conducted in animal preclinical models. There is a lack of human data examining these specific markers, as most studies on PRP in the human endometrium focus on clinical outcomes.

#### 3.3.2. Clinical Trials

The beneficial effects of PRP instillation in women with different endometrial pathologies have been described as different clinical results, with an increase in endometrial thickness in patients with TE [80,81] or RIF [82], even improving pregnancy results in these last patients [83].

However, there are not enough studies elucidating the possible mechanisms by which PRP produces its beneficial effect on human endometria. Wang et al. conducted a study to evaluate the in vitro effects of PRP on human endometrial mesenchymal stem cells (eMSC) and the in vivo effect on twenty patients with TE. eMSCs proliferation, growth and migration were significantly enhanced by PRP compared with the negative control in a dose-dependent manner and showed a tendency to increase cell adhesion. Following PRP infusion, women with TE had a significantly thicker endometrium, and their pregnancy rate increased to 60%. These results are in alignment with the previously cited studies where PRP proved to have similar effects in vitro and in vivo [84].

In order to comprehend the mechanisms by which PRP is able to improve endometrial receptivity in humans, gene expression studies in women with intrauterine adhesions (IUAs) have been undertaken. Chang et al. performed a time-series-based self-controlled study on six patients with IUAs treated with PRP and six control women, showing that receptivity gene expression after PRP was similar in both groups. A significant difference between the before and after PRP groups was found in genes related to immune response, showing an increase in pro-inflammatory factors in the endometrium. This study would indicate that the major effect of PRP in endometrial receptivity is assessing the adjustment of the immune environment [85].

Finally, work related to the effect of PRP in women with inflamed endometria has recently been reported. Boychuk et al. analysed 90 women who suffered from endometritis and divided them into two groups, one with conventional treatment based on classical schemes of combination of broad-spectrum antibiotics with metronidazole and/or clindamycin and the second group treated with PRP. Women in the PRP group presented increased clinical pregnancy rates in a year compared to the control group and the number of live births was two times higher [86].

Although these studies demonstrate positive effects and mechanisms of action of PRP on the endometrium, further research is needed due to inconsistent clinical outcomes—such as cases where endometrial thickness does not increase, or no significant clinical improvements are observed [87]. Moreover, the currently described mechanisms do not fully explain these discrepancies, highlighting the need for more comprehensive investigations to clarify PRP’s true therapeutic potential in endometrial repair.

## 4. Discussion

PRP demonstrates significant therapeutic potential in endometrial healing, inflammation modulation, and tissue regeneration, particularly in enhancing fertility outcomes. Preclinical models and in vitro studies have shown that PRP effectively reduces inflammation markers (e.g., MPO, NO, TNF-α, IL-1β, IL-6, and IL-8) and inhibits the TLR4/NF-κB pathway, while simultaneously activating the Nrf2/HO-1 pathway to reduce oxidative stress. Additionally, PRP promotes endometrial tissue repair, significantly elevating markers of cellular adhesion, proliferation, and regeneration (e.g., α-SMA, Cx-40, and E-Cad) in treated models. Transcriptomic studies on human endometrial stromal cells and other endometrial cell lines suggest that PRP fosters cell proliferation, migration, and matrix remodelling, as evidenced by the activation of MMP.

Clinical studies have reported improvements in endometrial thickness and pregnancy rates in PRP-treated women with TE or endometritis, further supporting PRP’s potential to enhance reproductive outcomes by modulating immune response and improving tissue receptivity.

Despite the promising findings from preclinical models demonstrating PRP’s anti-inflammatory and regenerative effects on the endometrium, significant gaps remain in fully understanding its therapeutic potential in humans. Current studies focus mainly on clinical outcomes, with limited insights into the specific molecular mechanisms and marker expressions in the human endometrium. Key areas for future research include validating the modulation of pathways like TLR4/NF-κB and Nrf2/HO-1 in human tissues, assessing the long-term safety and efficacy of PRP, and exploring its impact on endometrial receptivity and fertility restoration across diverse clinical contexts.

Looking ahead, combining PRP with other acellular therapies, such as exosomes, or cellular therapies, like MSCs, presents a promising avenue. The synergetic effects of these combined therapies have been explored in endometrial pathologies such as AS, where they have shown success in achieving pregnancy [88].

Addressing these research gaps is crucial to translating preclinical findings into standardized, evidence-based treatments for endometrial repair and fertility enhancement.

## 5. Conclusions

To fully understand PRP’s mechanisms of action in the human endometrium, more rigorous and controlled studies are essential. Future research should explore the potential of combining PRP with exosomes or mesenchymal stem cells to enhance endometrial regeneration and improve outcomes in patients with TE or AS.

Investigating the molecular pathways underlying PRP’s regenerative and anti-inflammatory properties will be crucial for validating its therapeutic potential. This will also be key to developing standardized protocols that can effectively benefit patients with impaired endometrial function.

## Abbreviations

**Abbreviation**	**Word**	**Abbreviation**	**Word**
α-SMA	Alpha-smooth muscle actin	L-PRP	Leukocyte- and platelet-rich plasma
ACD-A	Citrate dextrose A	L-PRF	Leukocyte- and platelet-rich fibrin
Adm	Adrenomedullin gen	LPS	Lipopolysaccharide
AS	Asherman syndrome	MCP-1	Monocyte chemoattractant protein 1
ATP/ADP	Adenosine Triphosphate/Adenosine Diphosphate	Mg^2+^	Magnesium Ion
Ca^2+^	Calcium Ion	MIP-1α	Macrophage Inflammatory Protein 1 Alpha
CaCl_2_	Calcium chloride	MMP-1, -2, -9	Matrix metalloproteinase 1, 2, and 9
CD40-L	CD40 Ligand	MPO	Mieloperoxidase
CCL	C-C motif chemokine ligands	MSCs	Mesenchymal stem cells
CK18	Cytokeratin 18	Mt2-MMP	Membrane type 2 matrix metalloproteinase gen
CK19	Cytokeratin 19	NAP-2	Neutrophil Activating Peptide 2
cKit-Mrna	c-Kit tyrosine kinase receptor messenger RNA	NF-κβ	Nuclear Factor Kappa B
CXCL	Chemokine C-X-C motif ligand	NO	Nitric oxide
CTGF	Connective Tissue Growth Factor	Nrf2	Nuclear factor erythroid 2-related factor 2
Cx40	Connexin 40	PDGF	Platelet-derived growth factor
Ecad	E-cadherin	PI3K	Phosphatidylinositol 3-kinase
ECM	Extracellular matrix	PF4	Platelet factor 4
EDTA	Ethylenediaminetetraacetic acid	PPP	Platelet-poor plasma
EGF	Epidermal growth factor	P-PRF	Platelet-rich fibrin
FGF	Fibroblast growth factor	PRGF	Plasma rich in growth factors
GFs	Growth factors	PRP	Platelet-rich plasma
HGF	Hepatocyte growth factor	P-PRP	Pure platelet-rich plasma
HO-1	Heme oxygenase 1	RANTES	Regulated upon activation, normal T cells expressed and secreted
HOXA10	Homeobox A10	RIF	Recurrent implantation failure
IGF-1	Insulin-like growth factor 1	SDF-1α	Stromal cell-derived factor 1 alpha
IGF-A	Insulin-like growth factor A	STAT3	Signal Transducer and Activator of Transcription 3
IL-1β	Interleukin 1 Beta	TE	Thin endometrium
IL-4	Interleukin 4	TGF-β	Transforming growth factor beta
IL-6	Interleukin 6	Timp1	Tissue Inhibitor of Metalloproteinases 1 gen
IL-8	Interleukin 8	TIMPs	Inhibitors of metalloproteinases
IL-10	Interleukin 10	TLR4	Toll-like receptor 4
IVF	In vitro fertilization	TLR4/NF-Κb	Toll-like receptor 4/nuclear factor κB
JAM-A, JAM-C	Junctional Adhesion Molecule A, C	TNF-α	Tumour necrosis factor-alpha
K^+^	Potassium Ion	TSP-1	Thrombospondin-1
Ki67	Ki-67 nuclear proliferation antigen gen	VEGF	Vascular endothelial growth factor
LFA-2	Lymphocyte Function-associated Antigen 2	vWF	von Willebrand factor
Lox	Lysil oxidase gen	ZO1	Zona occludens-1

## References

[1] Alves R., Grimalt R. (2018). A Review of Platelet-Rich Plasma: History, Biology, Mechanism of Action, and Classification. Ski. Appendage Disord..

[2] Martínez-Martínez A., Ruiz-Santiago F., García-Espinosa J. (2018). Platelet-rich plasma: Myth or reality?. Radiologia.

[3] Dohan Ehrenfest D.M., Rasmusson L., Albrektsson T. (2009). Classification of platelet concentrates: From pure platelet-rich plasma (P-PRP) to leucocyte- and platelet-rich fibrin (L-PRF). Trends Biotechnol..

[4] Delong J.M., Russell R.P., Mazzocca A.D. (2012). Platelet-rich plasma: The PAW classification system. Arthrosc. J. Arthrosc. Relat. Surg..

[5] Magalon J., Chateau A.L., Bertrand B., Louis M.L., Silvestre A., Giraudo L., Veran J., Sabatier F. (2016). DEPA classification: A proposal for standardising PRP use and a retrospective application of available devices. BMJ Open Sport Exerc. Med..

[6] Harrison P., Alsousou J., Andia I., Burnouf T., Dohan Ehrenfest D., Everts P., Langer H., Magalon J., Marck R., Gresele P. (2018). The use of platelets in regenerative medicine and proposal for a new classification system: Guidance from the SSC of the ISTH. J. Thromb. Haemost..

[7] Cavallo C., Roffi A., Grigolo B., Mariani E., Pratelli L., Merli G., Kon E., Marcacci M., Filardo G. (2016). Platelet-Rich Plasma: The Choice of Activation Method Affects the Release of Bioactive Molecules. BioMed Res. Int..

[8] Pachito D.V., Bagattini A.M., de Almeida A.M., Mendrone-Júnior A., Riera R. (2020). Technical Procedures for Preparation and Administration of Platelet-Rich Plasma and Related Products: A Scoping Review. Front. Cell Dev. Biol..

[9] Sweeny J., Grossman B.J., Brecher M., Krager S. (2017). Blood collection, storage, and component preparation methods. Technical Manual.

[10] Dashore S., Chouhan K., Nanda S., Sharma A. (2021). Preparation of platelet-rich plasma: National IADVL PRP taskforce recommendations. Indian Dermatol. Online J..

[11] Marques L.F., Stessuk T., Camargo IC C., Sabeh Junior N., Santos L.D., Ribeiro-Paes J.T. (2014). Platelet-rich plasma (PRP): Methodological aspects and clinical applications. Platelets.

[12] Kingsley C.S. (1954). Blood coagulation: Evidence of an antagonist to factor VI in platelet-rich human plasma. Nature.

[13] Everts P., Onishi K., Jayaram P., Lana J.F., Mautner K. (2020). Platelet-rich plasma: New performance understandings and therapeutic considerations in 2020. Int. J. Mol. Sci..

[14] Sánchez-González D.J., Méndez-Bolaina E., Trejo-Bahena N.I. (2012). Platelet-rich plasma peptides: Key for regeneration. Int. J. Pept..

[15] Mościcka P., Przylipiak A. (2021). History of autologous platelet-rich plasma: A short review. J. Cosmet. Dermatol..

[16] Flores J.R., Gallego M.A.P., García-Denche J.T. (2012). Plasma rico en plaquetas: Fundamentos biológicos y aplicaciones en cirugía maxilofacial y estética facial. Rev. Española Cirugía Oral Maxilofac..

[17] Gallo I., Sáenz A., Arévalo A., Roussel S., Pérez-Moreiras I., Artiñano E., Martínez-Peñuela A., Esquide J., Aspiroz A., Camacho I. (2013). Effect of autologous platelet-rich plasma on heart infarction in sheep. Arch. Cardiol. Mex..

[18] Hargrave B., Li F. (2012). Nanosecond pulse electric field activation of platelet-rich plasma reduces myocardial infarct size and improves left ventricular mechanical function in the rabbit heart. J. Extra-Corpor. Technol..

[19] Gawdat H.I., Tawdy A.M., Hegazy R.A., Zakaria M.M., Allam R.S. (2017). Autologous platelet-rich plasma versus readymade growth factors in skin rejuvenation: A split face study. J. Cosmet. Dermatol..

[20] Kao Y.C., Lin D.Z., Lee S.L., Chen C., Wang H.J., Chiu W.K. (2021). Assisted therapy with platelet-rich plasma for burn patients: A meta-analysis and systematic review. Burns.

[21] El Zouhbi A., Yammine J., Hemdanieh M., Korbani E.T., Nassereddine M. (2024). Utility of platelet-rich plasma therapy in the management of meniscus injuries: A narrative review. Orthop. Rev..

[22] Pham T.A.V., Tran T.T.P., Luong N.T.M. (2019). Antimicrobial effect of platelet-rich plasma against *Porphyromonas gingivalis*. Int. J. Dent..

[23] Sahli E., Özmert E., Günel M.D., Atilla H. (2024). Evaluation of the efficacy of subtenon autologous platelet-rich plasma therapy in patients with retinitis pigmentosa and factors affecting response to the treatment. Int. Ophthalmol..

[24] Alio J.L., Arnalich-Montiel F., Rodriguez A.E. (2012). The role of “eye platelet rich plasma” (E-PRP) for wound healing in ophthalmology. Curr. Pharm. Biotechnol..

[25] Sharara F.I., Lelea L.L., Rahman S., Klebanoff J.S., Moawad G.N. (2021). A narrative review of platelet-rich plasma (PRP) in reproductive medicine. J. Assist. Reprod. Genet..

[26] Teh W.T., McBain J., Rogers P. (2016). What is the contribution of embryo-endometrial asynchrony to implantation failure?. J. Assist. Reprod. Genet..

[27] Kasius A., Smit J.G., Torrance H.L., Eijkemans M.J.C., Mol B.W., Opmeer B.C., Broekmans F.J.M. (2014). Endometrial thickness and pregnancy rates after IVF: A systematic review and meta-analysis. Hum. Reprod. Update.

[28] Gharibeh N., Aghebati-Maleki L., Madani J., Pourakbari R., Yousefi M., Ahmadian Heris J. (2022). Cell-based therapy in thin endometrium and Asherman syndrome. Stem Cell Res. Ther..

[29] Liu K.E., Hartman M., Hartman A. (2019). Management of thin endometrium in assisted reproduction: A clinical practice guideline from the Canadian Fertility and Andrology Society. Reprod. BioMedicine Online.

[30] Eftekhar M., Tabibnejad N., Tabatabaie A.A. (2018). The thin endometrium in assisted reproductive technology: An ongoing challenge. Middle East Fertil. Soc. J..

[31] Wang Y., Tang Z., Teng X. (2024). New advances in the treatment of thin endometrium. Front. Endocrinol..

[32] Chang Y., Li J., Wei L.N., Pang J., Chen J., Liang X. (2019). Autologous platelet-rich plasma infusion improves clinical pregnancy rate in frozen embryo transfer cycles for women with thin endometrium. Medicine.

[33] Agarwal M., Mettler L., Jain S., Meshram S., Günther V., Alkatout I. (2020). Management of a thin endometrium by hysteroscopic instillation of platelet-rich plasma into the endomyometrial junction: A pilot study. J. Clin. Med..

[34] Efendieva Z., Vishnyakova P., Apolikhina I., Artemova D., Butov K., Kalinina E., Fedorova T., Tregubova A., Asaturova A., Fatkhudinov T. (2023). Hysteroscopic injections of autologous endometrial cells and platelet-rich plasma in patients with thin endometrium: A pilot randomized study. Sci. Rep..

[35] Puente Gonzalo E., Alonso Pacheco L., Vega Jiménez A., Vitale S.G., Raffone A., Laganà A.S. (2021). Intrauterine infusion of platelet-rich plasma for severe Asherman syndrome: A cutting-edge approach. Updates Surg..

[36] Aghajanova L., Cedars M.I., Huddleston H.G. (2018). Platelet-rich plasma in the management of asherman syndrome: Case report. J. Assist. Reprod. Genet..

[37] Rodríguez-Eguren A., Bueno-Fernandez C., Gómez-Álvarez M., Francés-Herrero E., Pellicer A., Bellver J., Seli E., Cervelló I. (2024). Evolution of biotechnological advances and regenerative therapies for endometrial disorders: A systematic review. Hum. Reprod. Update.

[38] Liu K., Cheng H., Guo Y., Liu Y., Li L., Zhang X. (2022). Autologous platelet-rich plasma intrauterine perfusion to improve pregnancy outcomes after implantation failure: A systematic review and meta-analysis. J. Obstet. Gynaecol. Res..

[39] Blair P., Flaumenhaft R. (2009). Platelet α-granules: Basic biology and clinical correlates. Blood Rev..

[40] Lin Y., Qi J., Sun Y. (2021). Platelet-rich plasma as a potential new strategy in the endometrium treatment in assisted reproductive technology. Front. Endocrinol..

[41] Thon J.N., Italiano J.E. (2012). Platelets: Production, morphology and ultrastructure. Handb. Exp. Pharmacol..

[42] Giannopoulou M., Dai C., Tan X., Wen X., Michalopoulos G.K., Liu Y. (2008). Hepatocyte growth factor exerts its anti-inflammatory action by disrupting nuclear factor-kappaB signaling. Am. J. Pathol..

[43] Lubkowska A., Dolegowska B., Banfi G. (2012). Growth factor content in PRP and their applicability in medicine. J. Biol. Regul. Homeost. Agents.

[44] Polasek J. (2005). Platelet secretory granules or secretory lysosomes?. Platelets.

[45] Amalia L. (2022). The role of platelet-selectin as a marker of thrombocyte aggregation on cerebral sinus venous thrombosis. J. Blood Med..

[46] Janus-Bell E., Mangin P.H. (2023). The relative importance of platelet integrins in hemostasis, thrombosis and beyond. Haematologica.

[47] Folkman J., Browder T., Palmblad J. (2001). Angiogenesis research: Guidelines for translation to clinical application. Thromb. Haemost..

[48] Chen Y., Yuan Y., Li W. (2018). Sorting machineries: How platelet-dense granules differ from α-granules. Biosci. Rep..

[49] Cognasse F., Laradi S., Berthelot P., Bourlet T., Marotte H., Mismetti P., Garraud O., Hamzeh-Cognasse H. (2019). Platelet inflammatory response to stress. Front. Immunol..

[50] Cleator J.H., Zhu W.Q., Vaughan D.E., Hamm H.E. (2006). Differential regulation of endothelial exocytosis of P-selectin and von Willebrand factor by protease-activated receptors and cAMP. Blood.

[51] Foster T.E., Puskas B.L., Mandelbaum B.R., Gerhardt M.B., Rodeo S.A. (2009). Platelet-rich plasma: From basic science to clinical applications. Am. J. Sports Med..

[52] Andia I., Sanchez M., Maffulli N. (2010). Tendon healing and platelet-rich plasma therapies. Expert Opin. Biol. Ther..

[53] Everts P.A.M., Knape J.T.A., Weibrich G., Schönberger J.P.A.M., Hoffmann J., Overdevest E.P., Box H.A.M., van Zundert A. (2006). Platelet-rich plasma and platelet gel: A review. J. Extra-Corpor. Technol..

[54] Cruz M.A., Diacovo T.G., Emsley J., Liddington R., Handin R.I. (2000). Mapping the glycoprotein Ib-binding site in the von Willebrand factor A1 domain. J. Biol. Chem..

[55] Giusti I., D’Ascenzo S., MacChiarelli G., Dolo V. (2020). In vitro evidence supporting applications of platelet derivatives in regenerative medicine. Blood Transfus..

[56] Middleton K.K., Barro V., Muller B., Terada S., Fu F.H. (2012). Evaluation of the effects of platelet-rich plasma (PRP) therapy involved in the healing of sports-related soft tissue injuries. Iowa Orthop. J..

[57] Andia I., Sánchez M., Maffulli N. (2012). Joint pathology and platelet-rich plasma therapies. Expert Opin. Biol. Ther..

[58] Flad H.-D., Brandt E. (2010). Platelet-derived chemokines: Pathophysiology and therapeutic aspects. Cell. Mol. Life Sci. CMLS.

[59] Dos Santos A.F., Rodrigues B.L., Mosaner T., Lana J.F. (2021). The regenerative mechanisms of platelet-rich plasma: A review. Cytokine.

[60] Vandercappellen J., Van Damme J., Struyf S. (2011). The role of the CXC chemokines platelet factor-4 (CXCL4/PF-4) and its variant (CXCL4L1/PF-4var) in inflammation, angiogenesis and cancer. Cytokine Growth Factor Rev..

[61] Wojdasiewicz P., Poniatowski Ł.A., Szukiewicz D. (2014). The role of inflammatory and anti-inflammatory cytokines in the pathogenesis of osteoarthritis. Mediat. Inflamm..

[62] Ferrante C.J., Leibovich S.J. (2012). Regulation of macrophage polarization and wound healing. Adv. Wound Care.

[63] Zhang J.-M., An J. (2007). Cytokines, inflammation, and pain. Int. Anesthesiol. Clin..

[64] Andia I., Abate M. (2013). Platelet-rich plasma: Underlying biology and clinical correlates. Regen. Med..

[65] Nurden A.T., Nurden P., Sanchez M., Andia I., Anitua E. (2008). Platelets and wound healing. Front. Biosci. J. Virtual Libr..

[66] Moog P., Kirchhoff K., Bekeran S., Bauer A.-T., von Isenburg S., Dornseifer U., Machens H.-G., Schilling A.F., Hadjipanayi E. (2020). Comparative Evaluation of the Angiogenic Potential of Hypoxia Preconditioned Blood-Derived Secretomes and Platelet-Rich Plasma: An In Vitro Analysis. Biomedicines.

[67] Peterson J.E., Zurakowski D., Italiano J.E., Michel L.V., Fox L., Klement G.L., Folkman J. (2010). Normal ranges of angiogenesis regulatory proteins in human platelets. Am. J. Hematol..

[68] Everts P.A., Ii G.F., Rothenberg J., Mautner K., Everts P.A., Ii G.F., Rothenberg J., Mautner K. (2020). The rationale of autologously prepared bone marrow aspirate concentrate for use in regenerative medicine applications. Regenerative Medicine.

[69] Martin P. (1997). Wound healing—Aiming for perfect skin regeneration. Science.

[70] Hayes A.J., Ralphs J.R. (2011). The response of foetal annulus fibrosus cells to growth factors: Modulation of matrix synthesis by TGF-β1 and IGF-1. Histochem. Cell Biol..

[71] Cáceres M., Martínez C., Martínez J., Smith P.C. (2012). Effects of platelet-rich and -poor plasma on the reparative response of gingival fibroblasts. Clin. Oral Implant. Res..

[72] Cui X., Ma Y., Wang H., Huang J., Li L., Tang J., Cheng B. (2021). The anti-photoaging effects of pre- and post-treatment of platelet-rich plasma on UVB-damaged HaCaT keratinocytes. Photochem. Photobiol..

[73] Hire J.M., Evanson J.L., Johnson P.C., Zumbrun S.D., Guyton M.K., McPherson J.C., Bojescul J.A. (2014). Variance of matrix metalloproteinase (MMP) and tissue inhibitor of metalloproteinase (TIMP) concentrations in activated, concentrated platelets from healthy male donors. J. Orthop. Surg. Res..

[74] Kuroda K., Matsumoto A., Horikawa T., Takamizawa S., Ochiai A., Kawamura K., Nakagawa K., Sugiyama R. (2023). Transcriptomic profiling analysis of human endometrial stromal cells treated with autologous platelet-rich plasma. Reprod. Med. Biol..

[75] Liu X., Wang Y., Wen X., Hao C., Ma J., Yan L. (2024). Platelet rich plasma alleviates endometritis induced by lipopolysaccharide in mice via inhibiting TLR4/NF-κB signaling pathway. Am. J. Reprod. Immunol..

[76] Zhang P., Li D., Yang Z., Xue P., Liu X. (2022). Nrf2/HO-1 pathway is involved the anti-inflammatory action of intrauterine infusion of platelet-rich plasma against lipopolysaccharides in endometritis. Immunopharmacol. Immunotoxicol..

[77] Jang H.Y., Myoung S.M., Choe J.M., Kim T., Cheon Y.P., Kim Y.M., Park H. (2017). Effects of autologous platelet-rich plasma on regeneration of damaged endometrium in female rats. Yonsei Med. J..

[78] Kshersagar J., Kawale A.A., Tardalkar K., Damle M.N., Chaudhari L.R., Sharma R., Joshi M.G. (2024). Activated platelet-rich plasma accelerate endometrial regeneration and improve pregnancy outcomes in murine model of disturbed endometrium. Cell Tissue Bank..

[79] Kim M.K., Yoon J.A., Yoon S.Y., Park M., Lee W.S., Lyu S.W., Song H. (2022). Human platelet-rich plasma facilitates angiogenesis to restore impaired uterine environments with asherman’s syndrome for embryo implantation and following pregnancy in mice. Cells.

[80] Molina A., Sánchez J., Sánchez W., Vielma V. (2018). Platelet-rich plasma as an adjuvant in the endometrial preparation of patients with refractory endometrium. JBRA Assist. Reprod..

[81] Zadehmodarres S., Salehpour S., Saharkhiz N., Nazari L. (2017). Treatment of thin endometrium with autologous platelet-rich plasma: A pilot study. JBRA Assist. Reprod..

[82] Kusumi M., Ihana T., Kurosawa T., Ohashi Y., Tsutsumi O. (2020). Intrauterine administration of platelet-rich plasma improves embryo implantation by increasing the endometrial thickness in women with repeated implantation failure: A single-arm self-controlled trial. Reprod. Med. Biol..

[83] Enatsu Y., Enatsu N., Kishi K., Otsuki J., Iwasaki T., Okamoto E., Kokeguchi S., Shiotani M. (2021). Clinical outcome of intrauterine infusion of platelet-rich plasma in patients with recurrent implantation failure. Reprod. Med. Biol..

[84] Wang X., Liu L., Mou S., Zhao H., Fang J., Xiang Y., Zhao T., Sha T., Ding J., Hao C. (2019). Investigation of platelet-rich plasma in increasing proliferation and migration of endometrial mesenchymal stem cells and improving pregnancy outcome of patients with thin endometrium. J. Cell. Biochem..

[85] Chang Y., Peng J., Zhu Y., Sun P., Mai H., Guo Q., Guo J., Liang X., Chen P. (2023). How platelet-rich plasma (PRP) intra-uterine injection improve endometrial receptivity of intrauterine adhesions in women: A time-series-based self-controlled study. J. Reprod. Immunol..

[86] Boychuk A.V., Kotsabyn N.V., Yakymchuk J.B., Nikitina I.M. (2024). Pregravid preparation of women with chronic endometritis in IVF cycles. Wiad. Lek..

[87] Aghajanova L., Sundaram V., Kao C.N., Letourneau J.M., Manvelyan E., Cedars M.I., Huddleston H.G. (2021). Autologous platelet-rich plasma treatment for moderate-severe Asherman syndrome: The first experience. J. Assist. Reprod. Genet..

[88] Tandulwadkar S., Mishra S., Gupta S. (2021). Successful application of combined autologous bone marrow-derived stem cells and platelet-rich plasma in a case of severe asherman syndrome and subsequent in vitro fertilization conception. J. Hum. Reprod. Sci..

